# Interferon-Induced Transmembrane Protein 3 rs34481144 C/T Genotype and Clinical Parameters Related to Progression of COVID-19

**DOI:** 10.1155/2023/2345062

**Published:** 2023-06-07

**Authors:** Melika Gholami, Fatemeh Sakhaee, Fahimeh Mirzaei Gheinari, Fattah Sotoodehnejadnematalahi, Morteza Ghazanfari Jajin, Mohammad Saber Zamani, Iraj Ahmadi, Enayat Anvari, Abolfazl Fateh

**Affiliations:** ^1^Department of Biology, Science and Research Branch, Islamic Azad University, Tehran, Iran; ^2^Department of Mycobacteriology and Pulmonary Research, Pasteur Institute of Iran, Tehran, Iran; ^3^Immunoregulation Research Center, Shahed University, Tehran, Iran; ^4^Department of Physiology, School of Medicine, Ilam University of Medical Science, Ilam, Iran; ^5^Microbiology Research Center (MRC), Pasteur Institute of Iran, Tehran, Iran

## Abstract

Recent research has associated the interferon-induced transmembrane protein 3 gene (IFITM3) with the outcomes of coronavirus disease 2019 (COVID-19), although the findings are contradictory. This study aimed to determine the relationship between *IFITM3* gene rs34481144 polymorphism and clinical parameters with COVID-19 mortality. The tetra-primer amplification refractory mutation system–polymerase chain reaction assay was used to analyze *IFITM3* rs34481144 polymorphism in 1,149 deceased and 1,342 recovered patients. The clinical parameters were extracted from the patients' medical records. In this study, the frequency of *IFITM3* rs34481144 CT genotypes (OR 1.47, 95% CI 1.23–1.76, *P* < 0.0001) in both sexes was significantly higher in deceased patients than in recovered patients. Moreover, *IFITM3* rs34481144 TT genotypes (OR 3.38, 95% CI 1.05–10.87, *P* < 0.0001) in women were significantly associated with COVID-19 mortality. The multivariable logistic regression model results indicated that mean age (*P* < 0.001), alkaline phosphatase (*P* = 0.005), alanine aminotransferase (*P* < 0.001), low-density lipoprotein (*P* < 0.001), high-density lipoprotein (*P* < 0.001), fasting blood glucose (*P* = 0.010), creatinine (*P* < 0.001), uric acid (*P* < 0.001), C-reactive protein (*P* = 0.004), 25-hydroxyvitamin D (*P* < 0.001), erythrocyte sedimentation rate (*P* < 0.001), and real-time PCR Ct values (*P* < 0.001) were linked with increased COVID-19 death rates. In conclusion, *IFITM3* rs34481144 gene polymorphism was linked to the mortality of COVID-19, with the rs34481144-T allele being especially important for mortality. Further studies are needed to confirm the results of this study.

## 1. Introduction

Coronavirus disease 2019 (COVID-19) is caused by severe acute respiratory syndrome coronavirus 2 (SARS-CoV-2), and it evolved into a global pandemic in 2020 [1]. As of March 2023, the COVID-19 prevalence had spread to almost every country in the world, and more than 6.8 million deaths were confirmed due to COVID-19 [2, 3]. The total death rate is around 2%, and 23% of COVID-19 patients have severe disease, necessitating intensive care and mechanical ventilation in 11% and 7%, respectively [4]. Severe COVID-19 is associated with acute renal damage, liver failure, cardiomyopathy, and coagulation malfunction. Consequently, it is imperative to identify proteins and genetic variables linked with COVID-19 susceptibility and outcome [5].

Interferon-induced transmembrane proteins (IFITMs) are a family of tiny proteins in animals with a single cell and are evolutionarily conserved among vertebrates. IFITMs can be found in a wide variety of organisms. The IFITM family in humans consists of five members such as immune-related IFITM1 and IFITM3, as well as unrelated IFITM5 and IFITM10. As essential gene for host immune system, IFITMs have developed under the selective pressure of infection by microorganisms [6]. IFITM proteins perform crucial functions in viral pathogenesis and are engaged in numerous facets of viral–host interaction. Single-nucleotide polymorphisms (SNPs) in the IFITM3 gene have been found in human populations, and some of these SNPs are linked to the severity and outcome of diseases caused by the influenza A virus (IAV) and other viruses [7, 8]. IFITMs have effectively suppressed various therapeutically significant human pathogenic viruses such as West Nile virus, dengue virus, Zika Virus, Ebola virus, SARS-CoV-2, and etc. [9].

Mechanistically, these SNPs either change the *IFITM3* expression or lead to the development of the N-terminally shortened IFITM3 isoform, *Δ*21-IFITM3, having diminished antiviral effectiveness against many viruses [7].

Several recent meta-analyses have also revealed the useful relationship between *IFITM3* gene polymorphisms such as rs34481144 and influenza virus susceptibility and clinical outcomes [6, 10]. The *IFITM3* rs34481144 is in the 5′ untranslated region and the T allele has been correlated with high risk of severe IAV. The main mechanism seems to be the incomplete binding of transcription factors to the promoter with the T allele, which leads to a decrease in mRNA expression compared to the C allele. In addition, the *IFITM3* rs34481144 C > T change disrupts the methylation site, and T carriers have reduced CD8+ T cells in their airways during natural influenza infection. IFITM3 induces the accumulation of airway CD8+ T cells at mucosal sites, and reduced expression may lead to decreased CD8 levels in the airways and increased risk of severe IAV [7]. With regard to SARS-CoV-2, the impact of IFITMs on infection remains unclear, but may depend on cellular localization, as indicated for IFITM3 in humans and mice. The IFITMs expression in human lung cells promotes SARS-CoV-2 infection by a mechanism involving interactions between spike viral proteins and IFITM [11]. In the context of the pandemic, the correlation between *IFITM3* SNPs and COVID-19 results has gained positive attention. Recent clinical investigations have studied the connection between *IFITM3* gene polymorphisms and COVID-19 susceptibility and severity; however, the results have been inconsistent [12]. Therefore, this study investigated the impact of the *IFITM3* rs34481144 and clinical parameters on the likelihood of SARS-CoV-2 infection and COVID-19 mortality in Iranian patients.

## 2. Materials and Methods

### 2.1. Study Subjects

In agreement with our previous study that we evaluated the relationship between *IFITM3* rs12252 and rs6598045 polymorphisms and the mortality rate of COVID-19 [13, 14], patients were selected from Ilam University of Medical Science between January 11 and December 9, 2021. SARS-CoV-2 RNA was detected in all enrolled patients' pharyngeal swab specimens by real-time reverse transcription polymerase chain reaction (real-time RT-PCR).

This was a retrospective study. Out of 11,125 patients, 2,491 patients were eligible to participate in the study. All patients included in this study met the following conditions: (1) people had to agree to take part in this study; (2) the patients were of similar ethnicity and Iranian nationality; (3) the selected patients were from only a designated hospital and their real-time RT-PCR test was positive; (4) the patients had no underlying disease such as cancer, human immunodeficiency virus, heart disease, diabetes, liver disease, cystic fibrosis, kidney disease, pregnancy, obesity, chronic obstructive pulmonary disease; (5) patients with definite disease outcome, including discharge or death.

Among 2,491 patients, 1,342 patients were treated in an outpatient setting (recovered patients), whereas 1,149 patients needed to be admitted to the hospital (deceased patients).

Recovered patients presented with mild clinical manifestations such as fever and respiratory symptoms, and an X-ray or computed tomography (CT) revealed evidence of pneumonia.

The deceased patients had the following conditions: breathlessness with a respiratory rate below 30; less than 92% oxygen saturation levels at rest in a single finger of one arm; rapid progression of lesions exceeding 50% within 24–48 hr; shock; acute respiratory failure requiring assistance from mechanical ventilation; involvement of other organs and the need for intensive care in an intensive care unit (ICU).

All the recruited patients' demographic information, epidemiological and clinical features, and laboratory results were extracted from the electronic medical record system of the Ilam University of Medical Sciences using data collection forms.

### 2.2. DNA Extraction and IFITM3 rs34481144 Genotyping

Blood samples (10 mL) treated with ethylenediaminetetraacetic acid were obtained from participants. The genomic DNA was isolated from the leukocytes using High Pure PCR Template Preparation Kit (Roche Diagnostics Deutschland GmbH, Mannheim, Germany), according to the manufacturer's instructions. The yield and purity of DNAs were measured using Nanodrop spectrophotometers (Thermo Scientific, USA), and the DNAs' quality was confirmed using electrophoresis gel.

The tetra-primer amplification refractory mutation system–polymerase chain reaction (T-ARMS–PCR) approach was used for genotyping *IFITM3* rs34481144 and also PRIMER1 software was used to design T-ARMS–PCR primer pairs for *IFITM3* rs34481144 (http://primer1.soton.ac.uk/primer1.html). The mismatched nucleotide was added to the primers at the third position from the 3′-end to facilitate accurate discrimination between two alleles (*Supplementary 2*).

T-ARMS–PCR was carried out in a total of 25 *μ*L volume, including 10 *μ*L TEMPase Hot Start DNA Polymerase (Ampliqon, Hamburg, Germany), 1.5 *µ*L of 5.0 pmol of outer forward primer, 2.0 *µ*L of 5.0 pmol of outer reverse primer, 2.5 *µ*L of 10 pmol of inner forward primer, 3.5 *µ*L of 10 pmol of inner reverse primer, 1 *μ*L (50 ng) of genomic DNA template, and 5.5 *μ*L of ddH_2_O. The conditions for PCR started with the initial denaturation at 95°C for 20 min followed by 40 cycles of 95°C for 20 s, 59°C for 15 s, and 72°C for 30 s, with a final extension at 72°C for 10 min. The PCR results were visualized by electrophoresis on a 3% agarose gel (*Supplementary 1*).

DNA sequencing using ABI 3500 DX Genetic Analyzer (ABI, Thermo Fisher Scientific, Waltham, MA, USA) was used to confirm the T-ARMS–PCR results (*Supplementary 1*).

### 2.3. Statistical Analyses

The statistical analysis was conducted using SPSS version 22.0 (SPSS, Inc., Chicago, IL, USA). The *χ*^2^ and Fisher's exact tests were utilized to assess the correlations between genotype and allele frequencies and COVID-19 mortality. The Mann–Whitney *U* test was used for the comparison of continuous variables expressed as mean ± standard division (SD). Multivariate models based on logistic regression were used to identify independent determinants of the possibility and mortality of COVID-19. The odds ratio (OR) and corresponding confidence interval (CI) of 95% were determined. The effect of *IFITM3* rs34481144 on COVID-19 mortality was evaluated using an area under the receiver operating characteristic curve (AUC–ROC) analysis. All of the tests were two sided, and statistical significance was defined as a *P*-value of less than 0.05.

Associations between *IFITM3* rs34481144 and COVID-19 mortality under five inheritance models (codominant, dominant, recessive, overdominant, and allele model) and Hardy–Weinberg equilibrium (HWE) were determined using the online SNPStats tool. Akaike information criterion (AIC) and Bayesian information criterion (BIC) were utilized to determine the best model (http://bioinfo.iconcologia.net/SNPStats).

## 3. Results

### 3.1. Characteristics of the COVID-19 Subjects

The clinical and laboratory characteristics of COVID-19 patients are indicated in Table 1. The present study included 2,491 COVID-19 patients, 1,149 in the deceased group and 1,342 in the recovered group. We showed clear evidence of an association between age and illness mortality. High levels of fasting blood glucose (FBS), liver enzymes profile, erythrocyte sedimentation rate (ESR), C-reactive protein (CRP), and creatinine (Cr) and low levels of lipid profiles, real-time PCR Ct value, uric acid, and 25-hydroxyvitamin D contributed to increased disease mortality.

### 3.2. Correlation between COVID-19 Mortality and IFITM3 rs34481144

**Figure 1:**
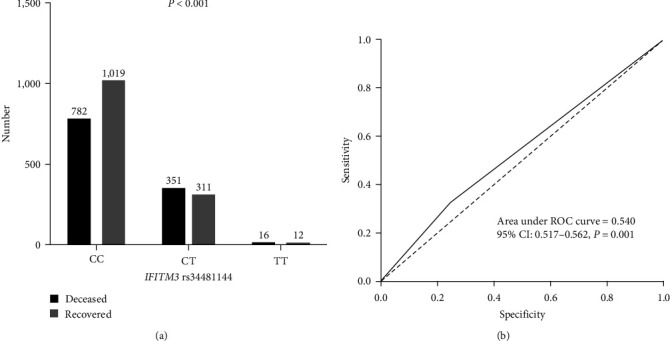
(a) Frequency of *IFITM3* rs34481144 in COVID-19 patients. (b) Receiver operating characteristic (ROC) curve with the *IFITM3* rs34481144 for prediction the mortality rate in COVID-19 patients.

The impact of *IFITM3* rs34481144 on COVID-19 mortality is depicted in Figure 1. Patients with *IFITM3* rs34481144 CT genotypes had significantly higher COVID-19 mortality compared to patients with other genotypes; however, COVID-19-recovered patients had CC genotypes (Figure 1(a)).

Under the inheritance models, we found that the genotype and allele frequencies of *IFITM3* rs34481144 CT were different in the two groups of SARS-CoV-2 patients. The best-fitting inheritance model for *IFITM3* rs34481144 was codominant with the lowest AIC and BIC values. The CT genotype (OR 1.47, 95% CI 1.23–1.76, *P* < 0.0001) was linked to a greater risk of death (Table 2).

When we stratified the analysis according to gender and *IFITM3* rs34481144 polymorphism frequency, COVID-19 mortality was correlated with *IFITM3* rs34481144 CT (OR 1.50, 95% CI 1.16–1.95) and TT genotypes (OR 3.38, 95% CI 1.05–10.87) in female, but was correlated with *IFITM3* rs34481144 CT genotype (OR 1.55, 95% CI 1.21–1.99) in male (Table 3).

*IFITM3* rs34481144 genotypes were incompatible with HWE in the recovered (*P* = 0.024) and deceased (*P* = 0.043) patients. It should be noted that the HWE may not be met in the case sample, indicating that the SNP is linked to the disease. Minor allele frequency (MAF) (T allele) in recovered, deceased, and all patients was 0.12, 0.17, and 0.14, respectively (Table 2).

Additionally, the AUC–ROC values for *IFITM3* rs34481144 were 0.540, showing that host genetic factors commonly influence viral infection mortality (Figure 1(b)).

### 3.3. Risk Factors Related to COVID-19 Mortality

The association of risk factors with COVID-19 clinical mortality was investigated using a multivariable logistic regression model. The mortality rate of COVID-19 infection was associated with mean age (OR 0.951, 95% CI 0.940–0.961, *P* < 0.001), alkaline phosphatase (ALP) (OR 0.998, 95% CI 0.996–0.999, *P* = 0.005), alanine aminotransferase (ALT) (OR 0.982, 95% CI 0.977–0.988, *P* < 0.001), low-density lipoprotein (LDL) (OR 1.016, 95% CI 1.013–1.019, *P* < 0.001), high-density lipoprotein (HDL) (OR 1.035, 95% CI 1.023–1.047, *P* < 0.001), FBS (OR 0.996, 95% CI 0.993–0.999, *P* = 0.010), Cr (OR 0.092, 95% CI 0.062–0.139, *P* < 0.001), uric acid (OR 1.928, 95% CI 1.769–2.101, *P* < 0.001), CRP (OR 0.982, 95% CI 0.976–0.988, *P* = 0.004), 25-hydroxyvitamin D (OR 1.044, 95% CI 1.032–1.055, *P* < 0.001), ESR (OR 0.970, 95% CI 0.962–0.978, *P* < 0.001), real-time PCR Ct values (OR 1.203, 95% CI 1.158–1.417, *P* < 0.001), and *IFITM3* rs34481144 CT (OR 0.499, 95% CI 0.376–0.662, *P* < 0.001) (Table 4).

## 4. Discussion

To our knowledge, this is the first comprehensive study in Iran on the correlation between *IFITM3* rs34481144 polymorphisms and mortality to COVID-19.

Based on several previous studies, we found the rs34481144 T variant was related to an elevated risk of COVID-19 death. The MAF (T allele) for *IFITM3* rs34481144 in European (0.41), African (0.13), African American (0.14), Latin American (0.28), Asian (0.006), East Asian (0.009), South Asian (0.23), and other Asian (0.00) was reported in dbSNP the NCBI dbSNP database (https://www.ncbi.nlm.nih.gov/snp/rs34481144).

The *IFITM3* rs34481144 T allele frequency was higher in the deceased patients than in the recovered patients. This confirmed the observation that *IFITM3* rs34481144 T allele carriers were more likely to COVID-19 mortality, with the CC genotype acting as a protective factor. The *IFITM3* rs34481144 (G > A) mutant reduces mRNA synthesis and modifies the concentration level of IFITM3 in peripheral blood monocytes [15].

In agreement with our study, Cuesta-Llavona et al. [16] demonstrated that COVID-19-positive individuals had a considerably greater frequency of *IFITM3* rs34481144 T carriers. In contrast, the findings in a meta-analysis indicated that the *IFITM3* rs34481144 gene variation was not associated with COVID-19 susceptibility in any of the gene models or illness severity [12]. Another study found that a higher prevalence of *IFITM3* rs34481144 T allele carriers was not seen in “severe” COVID-19 patients [17].

Pati et al. [18] hypothesized, based on previous research, that the *IFITM3* rs3448114 gene polymorphism may be positively linked with COVID-19 susceptibility and mortality, even though there have been few publications on the relationship between *IFITM3* rs34481144 and COVID-19 susceptibility.

According to a genotyping analysis conducted on three independent patient cohorts, individuals who carried the *IFITM3* rs34481144-A allele were approximately 2.6 times more likely to have a bad outcome following influenza virus infection [19]. Interestingly, the *IFITM3* rs34481144 CC genotype was considerably more common in controls, implying a protective effect against the influenza virus [20].

There have been few investigations on other *IFITM3* SNPs in influenza infection. The most relevant study found a connection between the rs34481144 T allele and illness severity, which was associated with lowering *IFITM3* expression compared to the C allele [7]. These studies also discovered that influenza-infected carriers of the A allele exhibited less CD8+ T cells in their airways, which was consistent with an IFITM3-mediated increase in airway CD8+ T cells accumulation. Thus, IFITM3 may play a significant role in enhancing the persistence of immune cells at mucosal locations, and IFITM3 functional variations may contribute to viral infection risk and illness severity [7].

IFITM3 exhibits antiviral action against IAV, SARS coronavirus, and numerous other viruses and has emerged as a crucial innate immunological barrier against viral infections in vertebrates [21]. It has been suggested that IFITM3 may serve as an antiviral effector molecule by enhancing the “rigidity” of endosomal membranes to restrict viral fusion [22]. Recent investigations have shown that IFITM3 can suppress the endocytosis of SARS-CoV-2, preventing fusion with the endosomal membrane. Furthermore, the polymorphisms of *IFITM3* gene, such as rs12252 and rs34481144, may be closely related to selective SARS-CoV-2 infection [18, 23]. It seems that the rs34481144 genotype is resistant to influenza virus via upregulating the expression of IFITM3 protein, highlighting that genetic variation (G > A) may change IFITM3 expression to further interfere with SARS-CoV-2 resistance [16, 19]. Additionally, *IFITM3* rs6598045 and rs34481144 as promoter region variants, influence promoter activity by altering the binding affinities of the transcription factor II-I and CCCTC-binding factor, respectively. These two regulatory SNPs affected the transcriptional activity of the *IFITM3* gene and were linked to the severity and susceptibility of viral infections including COVID-19 infection and IAV [14, 24].

It has been reported that severe COVID-19 patients revealed the *IFITM3* expression, but not *IFITM1*. However, *IFITM3* was not associated with poor outcomes or mortality rate in COVID-19 cases. These results contradict previous data that IFITM proteins provide better protection against IAV than SARS-CoV-2. The differences in these studies could be influenced by polymorphisms in *IFITM* genes. As shown in genomic investigations, there is a strong association between certain SNPs within the *IFITM3* gene and mortality in different ethnic groups [25–27]. For example, the role of *IFITM3* rs12252 with infection severity has been shown in several studies [13, 28, 29].

In our previous study, the AUC–ROC value for *IFITM3* rs12252 was 0.672, whereas it is 0.54 for *IFITM3* rs34481144. It seems that the effect of polymorphism *IFITM3* rs12252 is more effective than *IFITM3* rs34481144 in COVID-19 infection [13].

Since the beginning of the COVID-19 pandemic, changes in the lipid profile have been recorded; most notably a drop in cholesterol levels [30]. The relationship between LDL and HDL levels and CRP levels is inverse, meaning that the lower the LDL or HDL level, the greater the CRP levels, as also shown in our study [31]. Lipids are required for viruses to cross the cell membrane of their host. In addition, it is known that viral infections affect lipid metabolism in a manner that promotes virus replication [32]. Viruses utilize and change lipid signaling and metabolism to facilitate viral replication, as lipids are the primary component of membranes and serve crucial functions as energy sources and intercellular signaling factors [33]. SARS-CoV-2 replication, which enters the cell through endocytosis and uses intracellular organelles to manufacture their various components, necessitates lipid resources. As a result, investigating how SARS-CoV-2 infection impacts lipid metabolism and profile could illuminate the link between lipid profiles and how the body reacts to inflammation during COVID-19 [34].

As also found in our study, COVID-19 individuals have elevated liver enzymes in a median of 15% and up to 58% of cases. Although increased aminotransferases, with AST and ALT typically one to two times the upper limit of normal in SARS-CoV-2 patients, are the most common patterns of liver enzyme abnormalities, the prognostic importance of aberrant liver biochemistries remains unknown. In addition to direct liver injury, related inflammatory responses, congestive hepatopathy, hepatic ischemia, drug-induced liver injury, and muscle breakdown are potential contributing etiologies for increased liver enzymes in patients with SARS-CoV-2 [35, 36].

In this study, lower level of uric acid was related to COVID-19 mortality. Low levels of serum uric acid are frequent among COVID-19 patients requiring hospitalization and are associated with disease severity and progression to respiratory failure necessitating invasive mechanical ventilation [37]. Because uric acid has been discovered to act as an endogenous modulator of innate immunity, it is tempting to believe that low levels of serum uric acid may have exacerbated the cytokine storm observed during COVID-19 [38].

In this study, low real-time PCR Ct values are correlated with risk of mortality in SARS-CoV-2 infection. During the ongoing COVID-19 pandemic, it has become necessary to monitor patients infected with SARS-CoV-2 using viral loads in different sample types by real-time RT-PCR. Several reports have demonstrated that low Ct PCR values are correlated with a higher risk of ICU hospitalization and eventually, death [39, 40]. However, these results need to be investigated further.

Our study has a number of limitations that should be taken into account. There were no healthy controls in our study who had never had COVID-19. Additionally, no information was available regarding the patients' prior vaccinations. Furthermore, just one population of the same ethnicity was studied. More research on the many ethnicities in Iran is needed in order to generalize the association between these two polymorphisms to the entire society.

In conclusion, the *IFITM3* rs34481144 gene polymorphism was linked to COVID-19 mortality, and the SNP rs34481144 CT variation in both sexes and rs34481144 TT exclusively in women were related to COVID-19 mortality. In addition, clinical markers such as lipid and liver enzymes profiles, Cr, FBS, CRP, ESR, uric acid, 25-hydroxyvitamin D, and real-time PCR Ct values were correlated with infection mortality. In the future, it will be essential to find out if *IFITM3* rs34481144 gene polymorphism can accurately be linked to COVID-19.

## Figures and Tables

**Table 1:** Comparison laboratory parameters between deceased and recovered patients infected with COVID-19.

Variables	Deceased patients (*n* = 1,149)	Recovered patients (*n* = 1,342)	*P*-value
Mean age ± SD	59.3 ± 10.9	50.9 ± 12.9	<0.001^*∗*^
Gender (male/female)	611/538 (53.2/46.8%)	697/645 (51.9/48.1%)	0.535
ALT, IU/L (mean ± SD) (reference range: 5–40)	44.5 ± 23.9	33.2 ± 24.3	<0.001^*∗*^
AST, IU/L (mean ± SD) (reference range: 5–40)	36.9 ± 13.0	31.3 ± 15.7	<0.001^*∗*^
ALP, IU/L (mean ± SD) (reference range: up to 306)	201.9 ± 66.3	171.0 ± 90.6	<0.001^*∗*^
Cholesterol, mg/dL (mean ± SD) (reference range: 50–200)	116.6 ± 38.9	121.8 ± 36.3	<0.001^*∗*^
TG, mg/dL (mean ± SD) (reference range: 60–165)	116.6 ± 41.7	131.5 ± 61.5	<0.001^*∗*^
LDL, mg/dL (mean ± SD) (reference range: up to 150)	70.2 ± 35.6	107.1 ± 49.3	<0.001^*∗*^
HDL, mg/dL (mean ± SD) (reference range: >40)	30.6 ± 10.9	34.4 ± 11.6	<0.001^*∗*^
WBC, 10^9^/L (mean ± SD) (reference range: 4,000–10,000)	7,544.4 ± 2,663.4	7,717.4 ± 2,897.6	0.347
CRP, mg/L (mean ± SD) (reference range: <10 mg/L Negative)	67.3 ± 21.4	56.9 ± 20.9	<0.001^*∗*^
ESR, mm/1st hr (mean ± SD) (reference range: 0–15)	54.5 ± 15.6	46.5 ± 15.3	<0.001^*∗*^
FBS, mg/dL (mean ± SD) (reference range: 70–100)	110.7 ± 42.9	105.6 ± 41.3	<0.001^*∗*^
Platelets × 1,000/cumm (mean ± SD) (reference range: 140,000–400,000)	186 ± 76	185 ± 65	0.206
Uric acid, mg/dL (mean ± SD) (reference range: 3.6–6.8)	3.9 ± 1.2	5.6 ± 1.4	<0.001^*∗*^
Cr, mg/dL (mean ± SD) (reference range: 0.6–1.4)	1.2 ± 0.3	0.7 ± 0.2	<0.001^*∗*^
T3, ng/dL (mean ± SD) (reference range: 2.3–4.2)	3.1 ± 1.2	2.5 ± 0.8	0.061
T4, mcg/dL (mean ± SD) (reference range: 5.6–13.7)	8.9 ± 4.6	8.3 ± 3.3	0.091
TSH, mu/L (mean ± SD) (reference range: 0.4–4.5)	3.5 ± 1.4	3.3 ± 1.2	0.384
25-Hydroxy vitamin D, ng/mL (mean ± SD) (sufficiency: 21–150)	25.3 ± 10.1	35.9 ± 13.4	<0.001^*∗*^
Real-time PCR Ct values	13.1 ± 5.7	28.1 ± 8.1	0.007^*∗*^

ALT, alanine aminotransferase; AST, aspartate aminotransferase; ALP, alkaline phosphatase; TG, triglyceride; LDL, low-density lipoprotein; HDL, high-density lipoprotein; WBC, white blood cells; CRP, C-reactive protein; ESR, erythrocyte sedimentation rate; FBS, fasting blood glucose; T3, triiodothyronine; T4, thyroxine; TSH, thyroid-stimulating hormone; Ct, cycle threshold; SD, standard deviation; Cr, creatinine.  ^*∗*^Statistically significant (<0.05).

**Table 2:** *IFITM3* rs34481144 association with COVID-19 mortality.

		Groups					
Model	Genotype	Recovered patients	Deceased patients	OR (95% CI)	*P*-value	AIC	BIC
Allele	C	2,349 (88.0%)	1,915 (83.0%)	1.00	–	–	–
	T	335 (12.0%)	383 (17.0%)	1.98 (1.32–2.86)	<0.0001^*∗*^	–	–

Codominant	C/C	1,019 (75.9%)	782 (68.1%)	1.00	<0.0001^*∗*^	3,423.2	3,434.8
	C/T	311 (23.2%)	351 (30.6%)	1.47 (1.23–1.76)			
	T/T	12 (0.9%)	16 (1.4%)	1.74 (0.82–3.69)			

Dominant	C/C	1,019 (75.9%)	782 (68.1%)	1.00	<0.0001^*∗*^	3,425.9	3,442.5
	C/T-T/T	323 (24.1%)	367 (31.9%)	1.48 (1.24–1.77)			

Recessive	C/C-C/T	1,330 (99.1%)	1,133 (98.6%)	1.00	0.24	3,440.9	3,452.6
	T/T	12 (0.9%)	16 (1.4%)	1.57 (0.74–3.32)			

Overdominant	C/C-T/T	1,031 (76.8%)	798 (69.5%)	1.00	<0.0001^*∗*^	3,425.1	3,436.7
	C/T	311 (23.2%)	351 (30.6%)	1.46 (1.22–1.74)			

Minor allele frequency (T)		0.12	0.17	–	–	–	–

*IFITM3*, interferon-induced transmembrane protein 3; OR, odds ratios; CI, confidence intervals; AIC, Akaike information criterion; BIC, Bayesian information criterion.  ^*∗*^Statistically significant (<0.05).

**Table 3:** *IFITM3* rs34481144 and sex cross-classification interaction.

	Female			Male		
Genotype	Recovered patients	Deceased patients	OR (95% CI)	Recovered patients	Deceased patients	OR (95% CI)
CC	495	366	1.00	524	416	1.07 (0.89–1.29)
CT	146	162	1.50 (1.16–1.95)	165	186	1.55 (1.21–1.99)
TT	4	10	3.38 (1.05–10.87)	8	6	1.01 (0.35–2.95)

*IFITM3*, interferon-induced transmembrane protein 3; OR, odds ratios; CI, confidence intervals.

**Table 4:** Factors associated with deceased patients infected with COVID-19.

Factors		
Baseline predictors	OR (95% CI)	*P*-value
Mean age ± SD	0.951 (0.940–0.961)	<0.001^*∗*^
ALP, IU/L	0.998 (0.996–0.999)	0.005^*∗*^
ALT, IU/L	0.982 (0.977–0.988)	<0.001^*∗*^
HDL, mg/dL	1.035 (1.023–1.047)	<0.001^*∗*^
LDL, mg/dL	1.016 (1.013–1.019)	<0.001^*∗*^
FBS, mg/dL	0.996 (0.993–0.999)	0.010^*∗*^
Uric acid, mg/dL	1.928 (1.769–2.101)	<0.001^*∗*^
Cr, mg/dL	0.092 (0.062–0.139)	<0.001^*∗*^
ESR, (mm/1st hr)	0.970 (0.962–0.978)	<0.001^*∗*^
CRP, mg/L	0.982 (0.976–0.988)	0.004^*∗*^
25-hydroxyvitamin D, (ng/Ml)	1.044 (1.032–1.055)	<0.001^*∗*^
Real-time PCR Ct values	1.203 (1.158–1.417)	<0.001^*∗*^
*IFITM3* rs34481144 (CT)	0.499 (0.376–0.662)	<0.001^*∗*^

HDL, high-density lipoprotein; LDL, low-density lipoprotein; ESR, erythrocyte sedimentation rate; CRP, C-reactive protein; AST, aspartate aminotransferase; Ct, cycle threshold; SD, standard deviation; OR, odds ratios; CI, confidence intervals; *IFITM3*, interferon-induced transmembrane protein 3; Cr, creatinine.  ^*∗*^Statistically significant (<0.05).

## Data Availability

All data generated or analyzed during this study are included in the published article and supplementary files.

## Supplementary Materials

Supplementary 1 The result of *IFITM3* rs34481144 genotyping with T-ARMS–PCR. Lane No. 1, is ladder 100 bp, Lane No. 2, is negative control, Lane No. 3, is genotype CT (449 bp, 334 bp, and 156 bp), Lane No. 4, is genotype CC (449 bp and 334 bp), and Lane No. 5 is genotype TT (449 bp and 156 bp). The sequencing results of *IFITM3* rs34481144 genotypes for confirming the T-ARMS–PCR method (10% of samples randomly were sequenced).

Supplementary 2 Sequences and melting temperatures of the *IFITM3* rs34481144 primers.
