# Supplementary figures and images for: Interferon-Induced Transmembrane Protein 3 rs34481144 C/T Genotype and Clinical Parameters Related to Progression of COVID-19
Source: J Immunol Res. 2023 Jun 7;2023:2345062. doi: 10.1155/2023/2345062 (PMC10266908; doi:10.1155/2023/2345062)

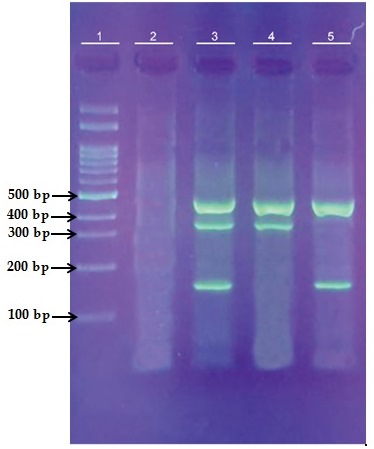


**Supplementary Figure 1:**


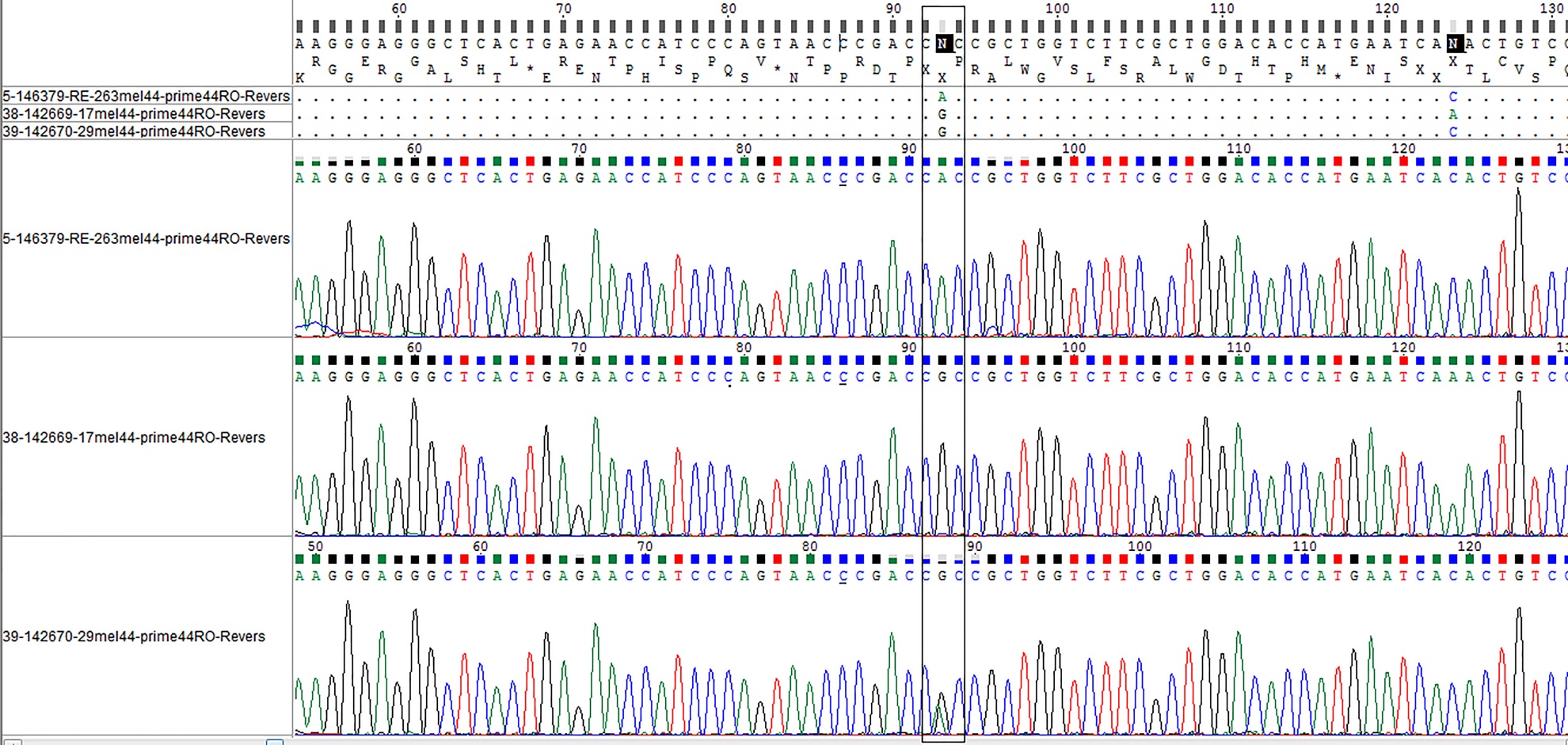


**Supplementary Figure 2:**

Supplement: Supplementary 1 — The result of IFITM3 rs34481144 genotyping with T-ARMS–PCR. Lane No. 1, is ladder 100 bp, Lane No. 2, is negative control, Lane No. 3, is genotype CT (449 bp, 334 bp, and 156 bp), Lane No. 4, is genotype CC (449 bp and 334 bp), and Lane No. 5 is genotype TT (449 bp and 156 bp). The sequencing results of IFITM3 rs34481144 genotypes for confirming the T-ARMS–PCR method (10% of samples randomly were sequenced). [file 2345062.f1.docx]
