# Supplementary material for: Interferon-Induced Transmembrane Protein 3 rs34481144 C/T Genotype and Clinical Parameters Related to Progression of COVID-19
Source: J Immunol Res. 2023 Jun 7;2023:2345062. doi: 10.1155/2023/2345062 (PMC10266908; doi:10.1155/2023/2345062)
Supplement: Supplementary 2 — Sequences and melting temperatures of the IFITM3 rs34481144 primers. [file 2345062.f2.docx]

**Supplementary Table 1:**

| **SNP** | **Primer** | **Primer sequence** | **size, bp** | **T_m_** | **Annealing Temp** |
| --- | --- | --- | --- | --- | --- |
| *IFITM3* rs34481144 (C/T) | OF | 5'-TCATCGGCACCCTCTGAGCATT-3' | - | 67.8 °C | 59.0 °C |
|  | OR | 5'-CCTCAGCAATTTGTTCCGCCCTC-3' | 449 | 67.8 °C |  |
|  | IF | 5'-TCCAGCGAAGACCAGCCG**T**-3' | 156 | 67.4 °C |  |
|  | IR | 5'-AGAACCATCCCAGTAACCCGAAC**G**-3' | 335 | 67.8 °C |  |

The specific nucleotides at 3′-end of primers are in bold, while the mismatches are underlined. SNP, single nucleotide polymorphism; OF, outer forward primer; OR, outer reverse primer; IF, inner forward primer; IR, inner reverse primer.
